# Engineering a 3D functional human peripheral nerve *in vitro* using the Nerve-on-a-Chip platform

**DOI:** 10.1038/s41598-019-45407-5

**Published:** 2019-06-20

**Authors:** Anup D. Sharma, Laurie McCoy, Elizabeth Jacobs, Hannah Willey, Jordan Q. Behn, Hieu Nguyen, Brad Bolon, J. Lowry Curley, Michael J. Moore

**Affiliations:** 1 AxoSim, Inc., New Orleans, LA USA; 2 Dept. of Biomedical Engineering, Tulane University, New Orleans, LA USA; 3 Brain Institute, Tulane University, New Orleans, LA USA; 4 GEMpath, Inc., Longmont, CO USA

**Keywords:** Peripheral nervous system, Translational research, Biomedical engineering

## Abstract

Development of “organ-on-a-chip” systems for neuroscience applications are lagging due in part to the structural complexity of the nervous system and limited access of human neuronal & glial cells. In addition, rates for animal models in translating to human success are significantly lower for neurodegenerative diseases. Thus, a preclinical *in vitro* human cell-based model capable of providing critical clinical metrics such as nerve conduction velocity and histomorphometry are necessary to improve prediction and translation of *in vitro* data to successful clinical trials. To answer this challenge, we present an *in vitro* biomimetic model of all-human peripheral nerve tissue capable of showing robust neurite outgrowth (~5 mm), myelination of hNs by primary human Schwann cells (~5%), and evaluation of nerve conduction velocity (0.13–0.28 m/sec), previously unrealized for any human cell-based *in vitro* system. To the best of our knowledge, this Human Nerve-on-a-chip (HNoaC) system is the first biomimetic microphysiological system of myelinated human peripheral nerve which can be used for evaluating electrophysiological and histological metrics, the gold-standard assessment techniques previously only possible with *in vivo* studies.

## Introduction

Preclinical models of neurodegenerative diseases, including both animal and *in vitro* models, have failed to translate to human success as a significant proportion of clinical trials fail and less than 7% of neurological drugs reach the marketplace^1,2^. Reasons for lesser predictivity of animal models are primarily attributed to differences in the underlying biology of the disease in animal vs. humans^2,3^. The last decade has observed a rapid pace in the development of *in vitro* microphysiological systems, including organs-on-chips, for mimicking human tissue physiology and, thus, improving the predictivity of preclinical drug screens^4,5^ and reducing the high rate of late-stage clinical trial failure^6^. These human cells based biomimetic microphysiological systems are expected to bridge the gap between animal experimentation and predicting the efficacy of the drugs in humans. Current *in vitro* systems which use human cells are either hybrid, using a combination of human neurons with rat Schwann cells (rSCs)^7^, or are a randomly oriented dissociated milieu of neuronal-glial cells which does not mimic peripheral nerve physiology and function^8,9^. Due to the complexity of the nervous system and the neuron-glia interplay, *in vitro* neural microphysiological systems to evaluate potential human responses to novel therapeutic candidates lag those of other organs. Peripheral nerves, in particular, lack appropriate human-relevant *in vitro* models. Thus far, animal testing remains the gold standard as they are the only models capable of supporting the standard clinically relevant metrics used for assessing peripheral neurotoxicity, namely electrophysiological and histopathological data^10^.

In this study, we describe an *in vitro*, microengineered, biomimetic, all-human peripheral nerve (HNoaC) comprised of induced pluripotent stem cell (iPSC)-derived neurons (hNs) and primary human Schwann cells (hSCs) that can provide data suitable for integrated nerve conduction velocity (NCV) and histopathological assessments. This all-human system is a significant extension of our *in vitro* “Nerve-on-a-chip” (NoaC) platform previously developed using embryonic rat dorsal root ganglion (DRG) neurons and rat SCs^11^. Most of the previous work done in this field is largely based on animal cells and uses a similar strategy to meticulously isolate somas from the axons in order to measure the electrical function of the cells^12–14^. However, these systems were either developed in two dimensions or represented excised nerves, and thus were not capable of providing conventional histological data, which is important for understanding the neuronal function. To our knowledge, this combination of hNs and hSCs has not previously been achieved for any other 3D human stem cell-based *in vitro* neural system. This all human nerve model showed, for the first time, numerous aspects of peripheral nerve physiology including robust defined axonal outgrowth (~5 mm), evidence of human Schwann cell myelination of human iPSC-derived neurons, and evaluation of NCV testing in an *in vitro* system, similar to *in vivo* animal testing. This innovative HNoaC platform can be used to create a variety of nerves (motor, sensory etc.) in the future and has the potential to accelerate the field of human disease modeling, drug discovery, toxicity screening, and precision medicine.

## Materials and Methods

### Schwann cell culture

A T-75 culture flask (353136; Corning, Corning, NY) was prepared by coating with a sterile-filtered, 0.1% poly-L-ornithine (PLO; Sigma-Aldrich, St. Louis, MO) solution in sterile water (Sigma-Aldrich, St. Louis, MO). The flask was then washed four times with sterile water. 7.5 mL of 10 µg/mL Laminin (Sigma-Aldrich, St. Louis, MO) in phosphate-buffered saline (PBS; Caisson Labs, Smithfield, UT) was added to the flask, which was held at 4 °C overnight. The Laminin solution was aspirated, and 15 mL of culture medium was directly placed into the T-75 culture flask, which was then equilibrated in a 37 °C incubator before cell plating.

Human Schwann Cell medium was purchased from ScienCell (Carlsbad, CA). Human primary Schwann cells (cat. No. 1700; ScienCell) were received in a cryovial with reportedly more than 5 × 10^5^ cells/mL. The vial was removed from cryopreservation and thawed in a 37 °C water bath. The contents of the vial were dispensed evenly onto the PLO/Laminin-coated T-75 Flask. The culture was left undisturbed at 37 °C in a 5% CO_2_ atmosphere for at least 16 hrs to promote attachment and proliferation. Culture medium was changed every 24 hours. Upon reaching 80% confluency, the hSCs were passaged by using 3 mL of Accutase® (Sigma-Aldrich), which was added to the flask for 3 mins at 37 °C. Once cells detached completely, 8 mL of hSC medium was placed in the flask. The 11 mL solution of detached hSCs was moved to a 15 mL conical tube and spun at 200 × g (Eppendorf 5810 R centrifuge, 18 cm radius; Eppendorf, Hamburg, Germany) for 5 minutes at room temperature (RT, approximately 22 °C). The supernatant was aspirated, and the pellet was resuspended in 1 mL of hSC culture medium. The cells were counted using a conventional hemocytometer (Hausser Scientific, Horsham, PA).

### Motor neuron culture

iCell® Motor Neuron (hNs; FUJIFILM Cellular Dynamics, Inc) medium was prepared using 100 mL of iCell® Neurons Base Medium (FUJIFILM Cellular Dynamics, Inc, Madison, WI) supplemented with 2 mL of iCell® Neural Supplement A and 1 mL of iCell® Nervous System Supplement. To prepare for thawing hNs, hNs medium was warmed to RT and 1 mL of hNs medium was added to a sterile 50 mL conical tube. One vial of hNs was thawed in a 37 °C water bath for approximately 2 mins and 30 seconds. The vial contents were transferred to the 50 mL conical tube containing 1 mL of hNs medium, drop-wise with a swirling motion, to mix the cell solution completely and minimize osmotic shock on thawed cells. The cell vial was then rinsed with 1 mL of hNs medium and transferred to the 50 mL tube. The volume of the solution was then brought to 10 mL by slowly adding hNs medium to the 50 mL centrifuge tube dropwise (2–3 drops/sec) while swirling. The cell solution was then transferred to a 15 mL conical tube and centrifuged at 200 × g for 5 mins at RT. The supernatant was aspirated, and cells were resuspended in 1 mL of hNs medium by flicking the tube and then pipetting up and down 2–3 times. A 10 μL sample of cell solution then was taken to perform a cell count using a hemocytometer.

### Spheroid fabrication

A non-treated, clear, “U” round bottom, 96 well, spheroid microplate (4515; Corning) was used for both monocultures of hNs and hSCs as well as co-cultures of hNs/hSCs. The concentration in cells/μL of media was calculated for both hSCs and hNs to permit calculation of the volumes needed to produce spheroids of the following sizes and compositions: hNs mono-cultures - 100000, 75000, 50000, or 25000; hSCs mono-cultures - 75000, 50000, or 25000; and co-cultures, 75000 hNs with either 75000, 50000 or 25000 hSCs. The compositions were chosen to ensure we can fit the largest possible spheroid in the dimension of the dual-hydrogel system to obtain maximal axonal density in the channel, hence making electrophysiological recordings more reliable. The volume calculated was added to microwell plates, then the volume of each well was brought to 200 μL by adding media warmed to 37 °C. The spheroid microplate was then centrifuged at 200 × g for 5 mins and placed in a 37 °C incubator in a 5% CO_2_ atmosphere until spheroid formation was observed (typically around 48 h). Half of the hNs medium was replenished every other day by removing 95 μL and replacing with 100 μL of fresh, warmed (to 37 °C) hNs medium.

### Fabrication of 3D dual-hydrogel nerve growth constructs

**Figure 1:**
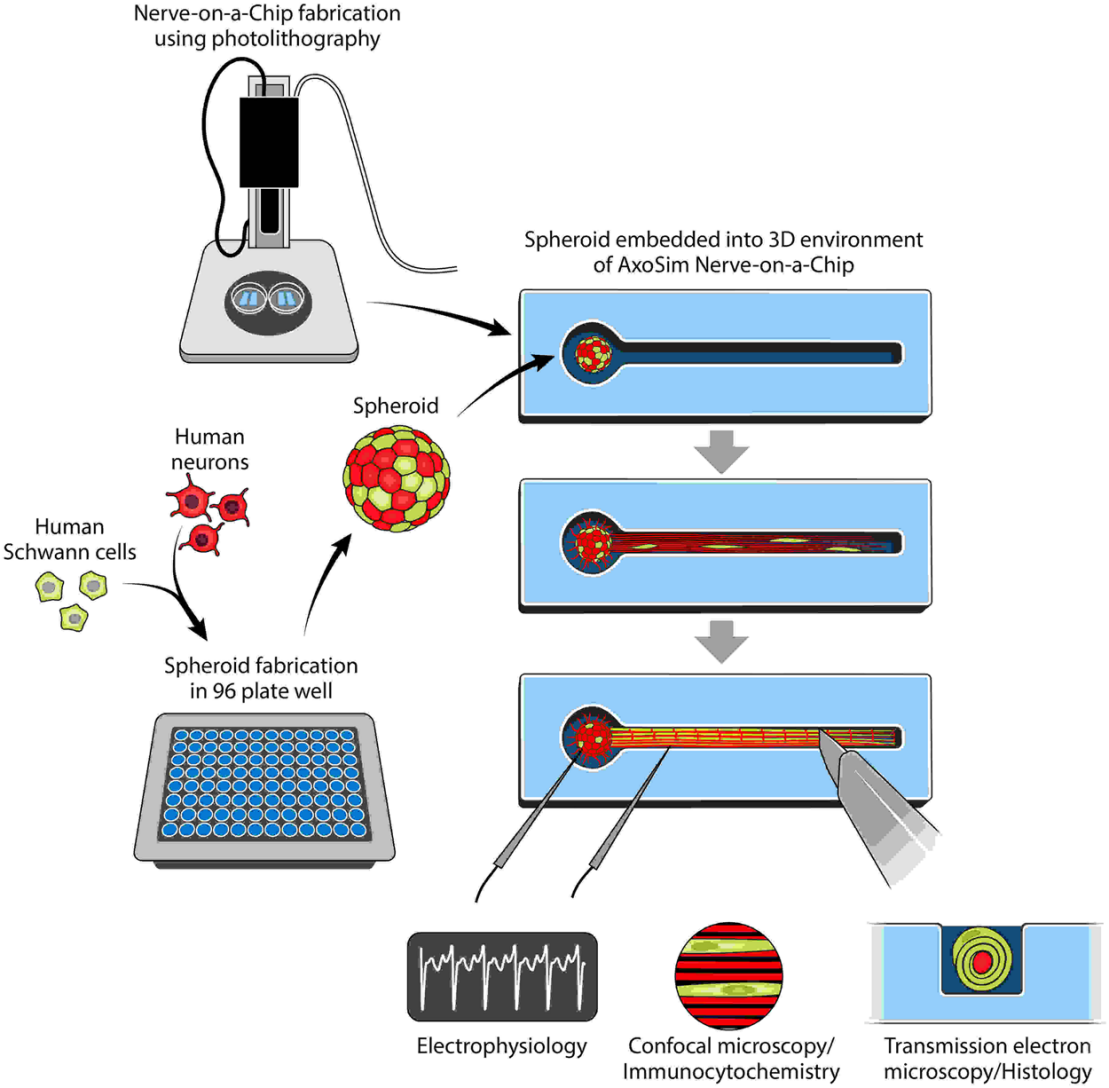
Study design showing the process of fabricating a Human Nerve-on-a-Chip (HNoaC) along with various means by which the system may be characterized. “*This figure is not covered by the CC BY licence. Credits to Anita Impagliazzo. All rights reserved, used with permission*”.

A dual-hydrogel scaffold was created on the membranes of Transwell® inserts (0.4 µm/PES; Corning) using a micro-photolithography technique similar to methods previously described^15^. All solutions were created with sterile-filtered PBS unless otherwise noted. The outer cell-restrictive (i.e., growth-resistant) photo-translinkable hydrogel was created using a solution of polyethylene glycol dimethacrylate 1000 (PEGDMA; Polysciences, Warrington, PA) and lithium phenyl-2,4,6-trimethylbenzoylphosphinate (LAP; Sigma Aldrich). First, 30% w/v PEGDMA and 1.1 mM LAP solutions were created and mixed in a 1:1 solution. The resulting 15% solution was sterile-filtered and added to Transwell® inserts placed in a volume of 0.6 mL while positioned under the lens of a Digital Micromirror Device (DMD, PRO4500 Wintech Production Ready Optical Engine; Wintech Digital Systems Technology Corp, Carlsbad, CA) on Rain-X (ITW Global Brands, Glenview, IL)-treated glass slides (Fig. 1). The mask and polymerization parameters were selected using commercial software (DLP Lightcrafter 4500 Control Software, Texas Instruments, Dallas, TX), and irradiation of the photo-translinkable solution occurred for 28–32 seconds using the ultraviolet light of 385 nm wavelength. After treatment, excess PEGDMA/LAP solution was removed from the insert and from within the void created by the photomask. The constructs were then washed using 2% Antibiotic/Antimycotic wash buffer (Thermo Fischer Scientific, Walton, MA) three times on the top and bottom of the insert for 10 minutes each. Wash buffer was removed from the insert and the inner keyhole-shaped channels. The void was carefully filled with 8% Growth Factor-Reduced Matrigel® Matrix (Corning) to create a cell-permeable scaffold and allowed to polymerize in a 37 °C incubator.

### Transferring spheroids to hydrogel construct

Two types of media were created using hNs medium (described above) to induce myelination in 3D constructs containing hNs. A Pre-myelination co-culture medium was created using hNs medium, 10% HyClone Characterized Fetal Bovine Serum (FBS; LaCell LLC, New Orleans, LA), and 1% Antibiotic-Antimycotic buffer. A Myelination co-culture medium was created with hNs medium, 10% FBS, 10 ng/mL of recombinant rat beta-Nerve Growth Factor (NGF; R&D Systems, Minneapolis, MN), and 50 μg/mL of L-ascorbic acid (Sigma-Aldrich). After formation, spheroids were transferred from the microplate using a pipette and placed onto a 35 mm tissue culture-treated dish (Cell Treat, Pepperell, MA) in a droplet of hNs medium. Spheroids were then placed into the 3D construct “bulb portion” (Fig. 1) within the Matrigel, using sterilized Dumont #5 fine-tipped forceps (11295–10; Dumont, Montignez, Switzerland). 1.5 mL of Pre-myelination medium was finally placed under the Transwell® membrane of the 6-well plates, and the loaded hydrogel constructs were placed in a 37 °C incubator in a 5% CO_2_ atmosphere for culture. Half changes of the medium were performed every other day. Both mono and co-culture spheroids were kept in the Pre-myelination co-culture medium for 1 week before being switched to Myelination co-culture medium for 3 weeks.

### Immunocytochemistry

Immunocytochemistry was performed to assess the 3D outgrowth of the neurons as well as migration of the Schwann cells. After four weeks in culture, half of the wells containing HNoaC systems were washed 3 times with PBS, fixed with 4% paraformaldehyde (PFA; pH 7.4; Electron Microscopy Sciences, Hatfield, PA) for 30 min at RT, and washed with PBS 4 times for 15 minutes each. Fixed samples were then placed in a 1X blocking solution containing PBS; 5% normal goat serum (Jackson ImmunoResearch, West Grove, PA); 0.2% Triton-X-100 (Sigma-Aldrich); and 0.4% bovine serum albumin (Sigma-Aldrich) for one hour at RT, followed by labeling with the following primary antibodies overnight in blocking solution at 4 °C: rabbit-α-s100 (ab868, 1:400; Abcam, Cambridge, MA); or mouse-α-βIII Tubulin (ab78078, 1:500; Abcam). Rabbit-α-myelin basic protein (MBP, ab133620, 1:500; Abcam) was also used in a separate trial under the same incubation conditions.

The following day, wells were washed with PBS 4 times for 8 min each at RT. The plate was then labeled with secondary antibodies, Alexa 488 goat anti-rabbit IgG (1:300, Abcam) or Alexa 568 goat anti-mouse IgG (1:300, Abcam), and DAPI (1:200, Sigma-Aldrich). Secondary antibodies and DAPI were dissolved in 1X blocker solution for 90 minutes in the dark at RT. The plate was washed 5 times, for 8 mins each, with PBS in the dark at RT. The plate was then parafilmed, foiled, and kept at 4 °C until microscopy was performed using a Nikon A1 confocal microscope (Nikon, Tokyo, Japan).

### Plastic resin embedding

All materials used for embedding were purchased from Electron Microscopy Sciences unless otherwise noted and were handled under a chemical flow hood with recommended personal protective equipment. Hydrogel constructs that were not used for immunocytochemistry were removed from culture and washed three times on both sides of the transmembrane well with PBS at RT prior to fixation. Plastic embedding is essential for evaluating the ultrastructure of the axons which is difficult to quantify by just performing immunostaining. The hydrogel constructs were then soaked in a solution of 4% PFA/0.5% glutaraldehyde for 30 minutes at RT. Secondary fixation and staining of cellular lipids was achieved by post-fixation with 1% osmium tetroxide in PBS, pH 7.4, for 2 hours under dark conditions at RT. The constructs were then washed with PBS 3 times for 15 minutes prior to counterstaining with 2% aqueous uranyl acetate for 30 minutes under dark conditions at RT. Dehydration was done with graded ethanol washes at RT, beginning with a 10-minute wash with 50% ethanol/PBS, a 10-minute wash with 70% ethanol/PBS and an overnight wash with 95% ethanol/PBS. The following day, the constructs were washed twice with 100% ethanol for 30 minutes at RT.

With a scalpel, hydrogel constructs were dissected individually from the transmembrane wells, without removal of the PEGDMA, under a dissecting microscope. Constructs were placed in Flat Embedding Molds (EMS 70902, Electron Microscopy Sciences). Remaining ethanol was given time to evaporate from the fixed hydrogels before replacement with infiltration medium consisting of a 1:1 mixture of Spurr’s resin (Low Viscosity Embedding Media Spurr’s Kit; Electron Microscopy Sciences) and propylene oxide. Infiltration medium was left for 75 minutes before it was replaced by 100% Spurr’s Resin, which was cured overnight in a 70 °C oven and for 48 more hours at RT before ultramicrotome sectioning.

### Sectioning and transmission electron microscopy (TEM)

Sectioning and TEM evaluation was performed at the Shared Instrumentation Facility (SIF) at Louisiana State University (Baton Rouge, LA). Ultrathin sections were cut to a thickness of 80–100 nm at four locations within the HNoaC specimen: within the bulb of the tissue, where the bulb met the channel and the proximal channel (i.e., near the bulb), and distal channel (Fig. 1). Sections were placed on Formvar carbon-coated copper grids, 200 mesh, and impregnated with metal by floating on droplets of 2% uranyl acetate for 20 mins at RT. They were then rinsed with deionized water droplets 3 times, for 1 min. To visualize, a JEOL 1400 TEM (Peabody, MA) was used with an accelerating voltage of 120 kV at varying magnifications.

### Histomorphometric analysis

Data acquired from TEM images of HNoaC cross sections included the following clinically-relevant metrics which are commonly associated with pathologies of the peripheral nervous system: myelinated and unmyelinated axon diameter, G-ratio (i.e., the ratio of the axon diameter to the diameter of the whole fiber [axon + myelin sheath]), and percent of myelinated fibers. All metrics were elucidated by two different, independent, blinded researchers by measuring both unmyelinated axons and axons encircled by 3 or more layers of dark myelin wrapping. G-ratio and axon diameter were measured using the scale, threshold and measure functions in Fiji^16^. Ten images taken from throughout the construct were randomly selected for measurement. First, myelinated fibers were identified as being surrounded by 3 or more myelin laminae, while unmyelinated fibers were either naked or engulfed by a Schwann Cell. The axon diameter of the unmyelinated fibers was measured using the threshold function to find the area of the fiber. Diameter was then calculated from this measurement. G-ratio calculation for myelinated fibers began by finding the inner axonal area in a similar fashion to axon diameter (thresholding the whole area to estimate the average diameter.) Second, the outer area was measured and included the dark myelin lamellae. The large nucleated bodies of Schwann cells were excluded when measuring the outer extent of the myelin sheath. The inner and outer diameters were then calculated from the area measurement. G-ratio, as described above, was calculated last. Percent myelination was also evaluated in Fiji by sampling 5 micrographs from the neck portion of the bulb that contained at least 1 myelinated fiber and 5 non-myelinated fibers. A simple tally of myelinated fibers observed, and total fibers observed was used to calculate percent myelination.

### Electrophysiology

After a month in co-culture, the Transwell® insert with the reconstituted nerve was placed on a stage for electrophysiological testing. Two tubes, one for dispensing and the other for aspiration, were placed along the edges of the Transwell® insert for perfusing oxygenated artificial cerebrospinal fluid (ACSF)^11^ over tissue samples. For recording compound action potentials (CAP), a pulled glass micropipette electrode (1–4 MΩ) was inserted into the bulb of the channel near the clustered cell bodies, and the axons growing in the channel were stimulated with a concentric bipolar platinum-iridium electrode positioned 1–3 mm distal to the bulb. A platinum recording electrode was placed in the ACSF-filled glass micropipette and was connected to an amplifier set at 100x gain and 0.1 Hz high pass to 3 kHz low pass filtering. Stimulation pulse height and width were kept at 10 volts and 200 µsec, respectively. Samples were stimulated at a maximum repeat rate of 1 Hz, and at least 50 stimulations were applied per sample. Using an analog-to-digital converter (PowerLab; AD Instruments, Colorado Springs, CO), CAP waveforms were visualized and further stored using LabChart software (AD instruments). After CAP recording, using a stereomicroscope and camera, a snapshot of the stimulating and recording electrodes were taken to determine the distance between them for NCV calculation. Latency was measured by subtracting the location of stimulus artifact by CAP peak location. NCV for myelinated hMN/hSC co-cultures and unmyelinated hMN monocultures was evaluated by dividing the distance between stimulating and recording electrodes by latency.

### Statistics

One-way analysis of variance (ANOVA) with Tukey *post-hoc* test was conducted using GraphPad Prism software (GraphPad Software, Inc., La Jolla, CA, USA) to evaluate size differences between different kind of spheroids. For analysis of electrophysiology, mean and standard deviation were calculated and an unpaired *t*-test was performed (GraphPad Software.) A p-value ≤ 0.05 was used to assign significant differences between the means. All the data was collected using at least three different replicates.

## Results

### Schwann cells enhanced assembly of neurons into spheroids

In order to create a spheroid which fits within the dimensions of the NoaC platform (i.e., less than 1,000 μM in diameter, with the maximum number of cells), we fabricated spheroids with different cellular densities. We also compared the sizes of different spheroids to understand the interactions between the hNs and hSCs.

**Figure 2:**
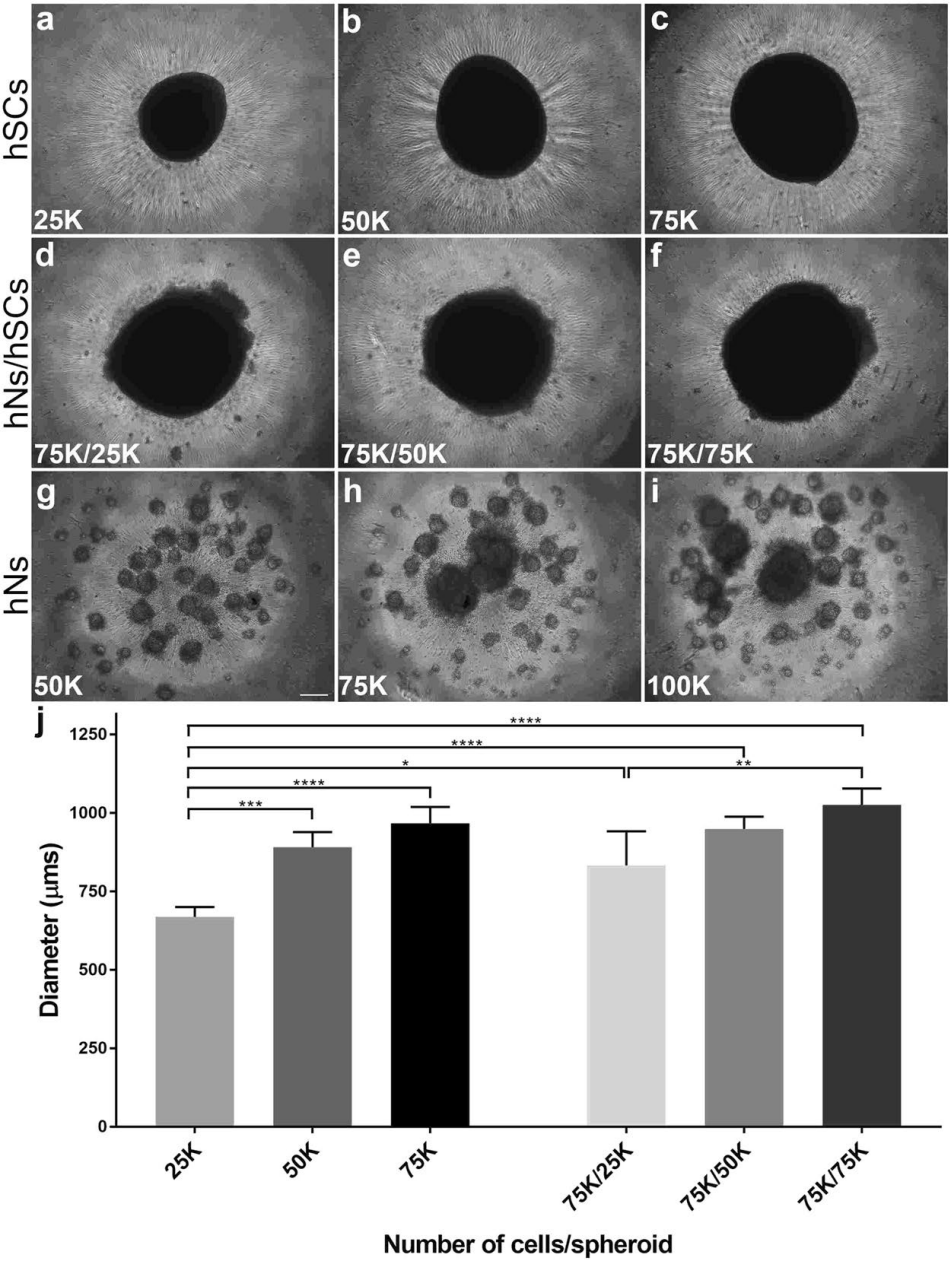
Fabrication of spheroids consisting of human neurons (hNs) and/or human Schwann cells (hSCs) after 2 days *in vitro*. Presence of hSCs expedited spheroid formation in the co-culture system while the hN-only condition did not form spheroids in 2 days. hSC spheroids were fabricated with three different cellular densities: 25,000 (**a**) 50,000 (**b**) and 75,000 (**c**). Co-culture spheroids were created with a constant hN density (75,000) but a changing hSC density: 25,000 (**d**) 50,000 (**e**) and 75,000. (**f**) In parallel, hN spheroids were fabricated at three different densities: 50,000 (**g**), 75,000 (**h**) and 100,000. (**i**,**j**) Comparison of diameters of different spheroids revealed that co-culture spheroids were more compact as compared to mono-culture spheroids, showing that the affinity of hSCs for hNs resulted in a more tightly packed cluster of cells. K (multiple of thousand). N = 4 and error bars represent standard error of the mean (SEM). Scale bar: 100 µm. (****p-value ≤ 0.0001), (***p-value ≤ 0.001), (**p-value ≤ 0.01), (*p-value ≤ 0.05).

**Figure 3:**
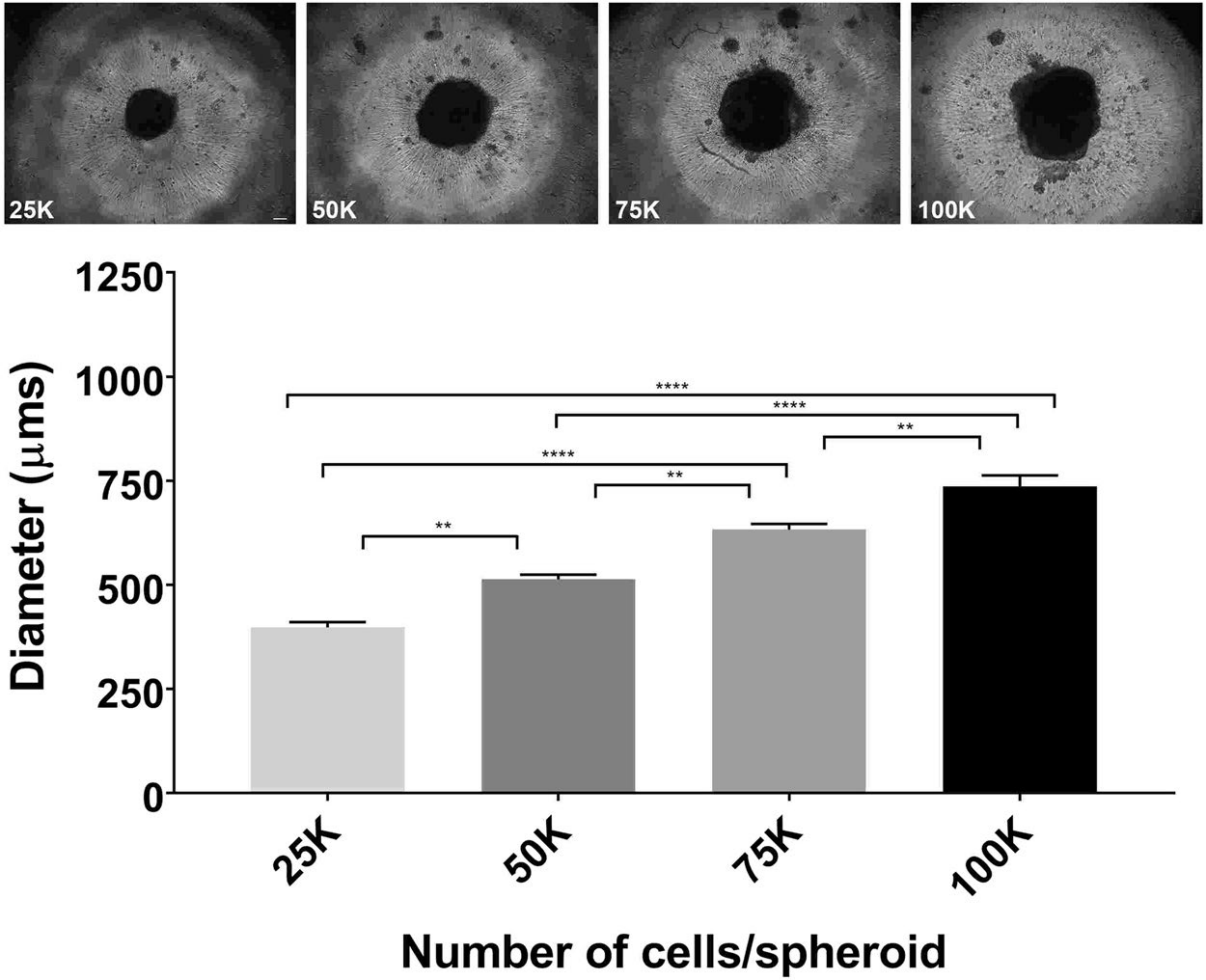
Fabrication of spheroids consisting of human neurons (hNs) alone. Qualitative inspection at > 2 days after initiating the hN-only culture showed a consistent significant increase in spheroid sizes as the total number of cells increase (25 K, 50 K, 75 K and 100 K). Graph comparing the individual spheroid diameters supported the qualitative inspection. K (multiple of thousand). N = 4 and error bars represent standard error of the mean (SEM). Scale bar: 100 µm. (****p-value ≤ 0.0001), (**p-value ≤ 0.01).

After placing the desired number of cells in low attachment, round-bottom plates, we monitored the formation of spheroids daily. Mono-cultures of hSCs formed spheroids within approximately 2 days and were found to be regular in shape with sharp edges (Fig. 2a–c). In contrast, monocultures of hNs did not self-assemble into spheroids in 2 days and instead formed numerous smaller spherical structures (Fig. 2g–i). In co-cultures, hSCs appeared to facilitate the incorporation of hNs into spheroids (~2 days) when compared to spheroids formed from hNs alone (~3–9 days, Fig. 3). The edges of the co-culture spheroids (Fig. 2d–f) were less sharply defined compared to spheroids containing the hSCs alone, possibly due to the non-homogeneous nature of the co-culture spheroid. Interestingly, the size of co-culture spheroids comprised of 75,000 hNs and 75,000 hSCs (1025 ± 52 µm) was found to be very similar to the size of 75,000 hSCs alone (967 ± 51 µm), suggesting that co-culture spheroids were more tightly packed and thereby confirming interactions between the two cell types.

By measuring the diameters of each of the different spheroid types (Figs 2 and 3), we determined that having 75,000 neurons is the optimal number of hNs for constituting the HNoaC system as the spheroid size was found to be about 833 ± 108, 948 ± 39 and 1025 ± 52 µm when we created co-culture spheroids with 25,000, 50,000 and 75,000 hSCs respectively. Neuron-only cultures revealed that spheroids increased in size predictably as the number of cells increased. Neurons only spheroids size was found to be 398 ± 26, 513 ± 22, 633 ± 27 and 736 ± 54 µm for 25,000, 50,000, 75,000 and 100,000 hNs respectively. All four hNs conditions (Fig. 3) saw a successive, significant increase in the spheroid size revealing that packing density across the four spheroids (25 K, 50 K, 75 K, and 100 K) does not vary substantially, and that the total number of cells appears to contribute to spheroid size more than the interaction between the various cell types in the spheroid.

### Co-culture spheroids showed robust neurite outgrowth in the NoaC system

While the outer portion of the dual hydrogel system was constructed with growth-resistant 15% PEGDMA, the inner part of the channel was filled with fully concentrated (8–12 mg/mL) Matrigel as a growth-promoting substrate. After gel formation, spheroids were gently transferred on top of the bulb part of the channel and left to grow in medium containing 10% FBS but lacking NGF. After a week, the incubation solution was switched to medium supplemented with NGF and L-ascorbic acid to facilitate neurite growth and myelination by the hSCs in contact with the growing axons.

**Figure 4:**
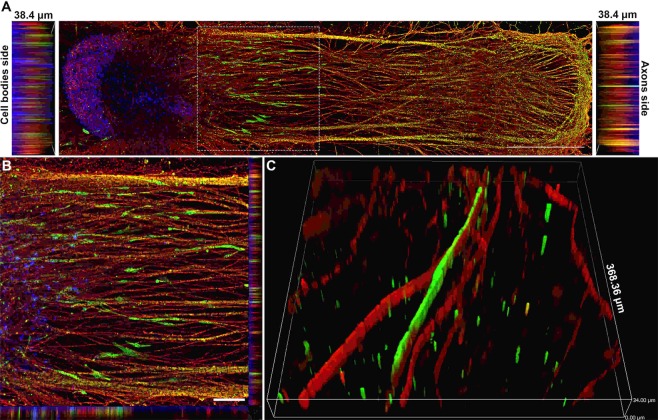
Schwann cells migrated out of the spheroid and elongated along the axons. (**A**) Image showing how human Schwann cells (hSCs) stained for the hSC marker S100 (green) migrated out of the spheroid along with growing axons stained for βIII-tubulin (red) over a period of 4 weeks. Nuclei were labeled with DAPI (blue). Scale bar: 1000 µm. (**B**) High-magnification image of inset from image A. Scale bar: 25 µm. (**C**) 3D image showing close-up of the relationship between hSCs (green) and myelinated axons (red). Slice size was 368.36 × 368.36 × 34.00 µm.

Confocal imaging revealed the 3D nature of the reconstituted *in vitro* nerves and showed that both cell bodies and axons were present throughout the depth of the channel (Fig. 4). Neurites grew an average of ~1 mm every week (Supplemental Fig. 4). Calcein staining at 4 weeks’ time point revealed the viability of these constructs at the 4-week time point (Supplemental Fig. 5). The addition of FBS was a key factor in optimizing myelination as basal media with ascorbic acid minus FBS did not support hSC migration and myelination, and the inner hydrogel itself collapsed in the absence of FBS (Supplemental Fig. 3). It was surprising to see that FBS had no impact on the stabilization of hNs alone condition. However, FBS stabilized the co-culture system showing that FBS has an impact on Schwann cell-axonal interactions. Immunostaining after four weeks with S100 revealed that hSCs cells migrated about 1–1.5 mm outside the spheroid and elongated along the growing axons (Fig. 4A–C), while the axons reached the very end of the Matrigel-filled channel (~5 mm). Interestingly, for many co-culture samples, spheroids influenced the growth of axons such that they appeared to turn back after growing for some distance, possibly because of the chemotactic effect created by growth factors released from hSCs in the spheroid (Supplemental Fig. 2). The effect on axons was more prominent as the number of hSCs in the spheroid increased.

### Myelination and Nerve Fiber Structure of *in vitro* Human Nerves

**Figure 5:**
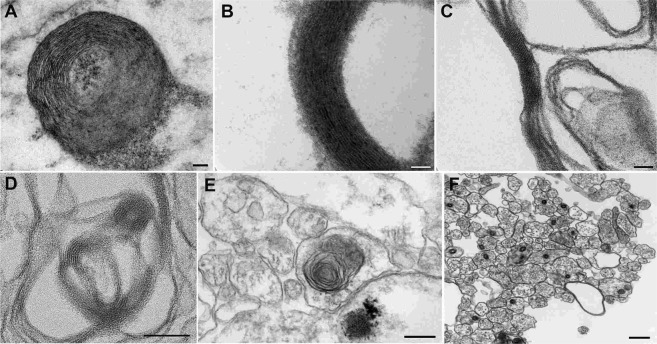
Various stages of myelin formation observed in *in vitro* reconstituted human nerve. (**A**) Non-compact myelin. (**B**) Compact myelin. (**C**) Myelin in the process of compaction. (**D**) Myelin formation without axons. (**E**) Intracytoplasmic lamellar bodies. (**F**) Naked (unmyelinated) axons.

Finally, along with immunostaining and confocal microscopy, we also performed plastic resin embedding and sectioning to evaluate the level and quality of myelination in the system with TEM. TEM micrographs from the bulb sections revealed the presence of both neuronal and Schwann cell bodies, however, micrographs from the channel sections demonstrated the presence of either Schwann cells or axons showing that neuronal bodies stayed at one location in the bulb (Supplemental Fig. 5). Evidence of effective myelination in the system included but was not limited to non-compacted myelin (Fig. 5A), compact myelin (Fig. 5B), and myelin in the process of compacting (Fig. 5C). For the axons, where we saw evidence of myelination, the G-ratio of myelinated nerve fibers was 0.57 ± 0.16. Axonal diameters of the myelinated and unmyelinated axons were 0.55 ± 0.33 and 0.40 ± 0.15 µm, respectively. Percent myelinated axons in the neck region of the channel were found to be 4.7%. We also saw evidence of laminar myelin formation without axons (Fig. 5D), the presence of intracytoplasmic lamellar bodies (Fig. 5E), and naked (unmyelinated) axons (Fig. 5F). The appearance of the intracytoplasmic lamellar bodies, which consisted of membrane whorls with relatively regular spacing, was interpreted to represent autophagosome production consistent with the recycling of senescent organelles. The distribution of lamellar bodies was sparse and apoptotic nuclei were not observed, indicating that affected cells were not engaged in programmed cell death.

### *In vitro* human nerves exhibit effective, composition-dependent electrical conductivity

**Figure 6:**
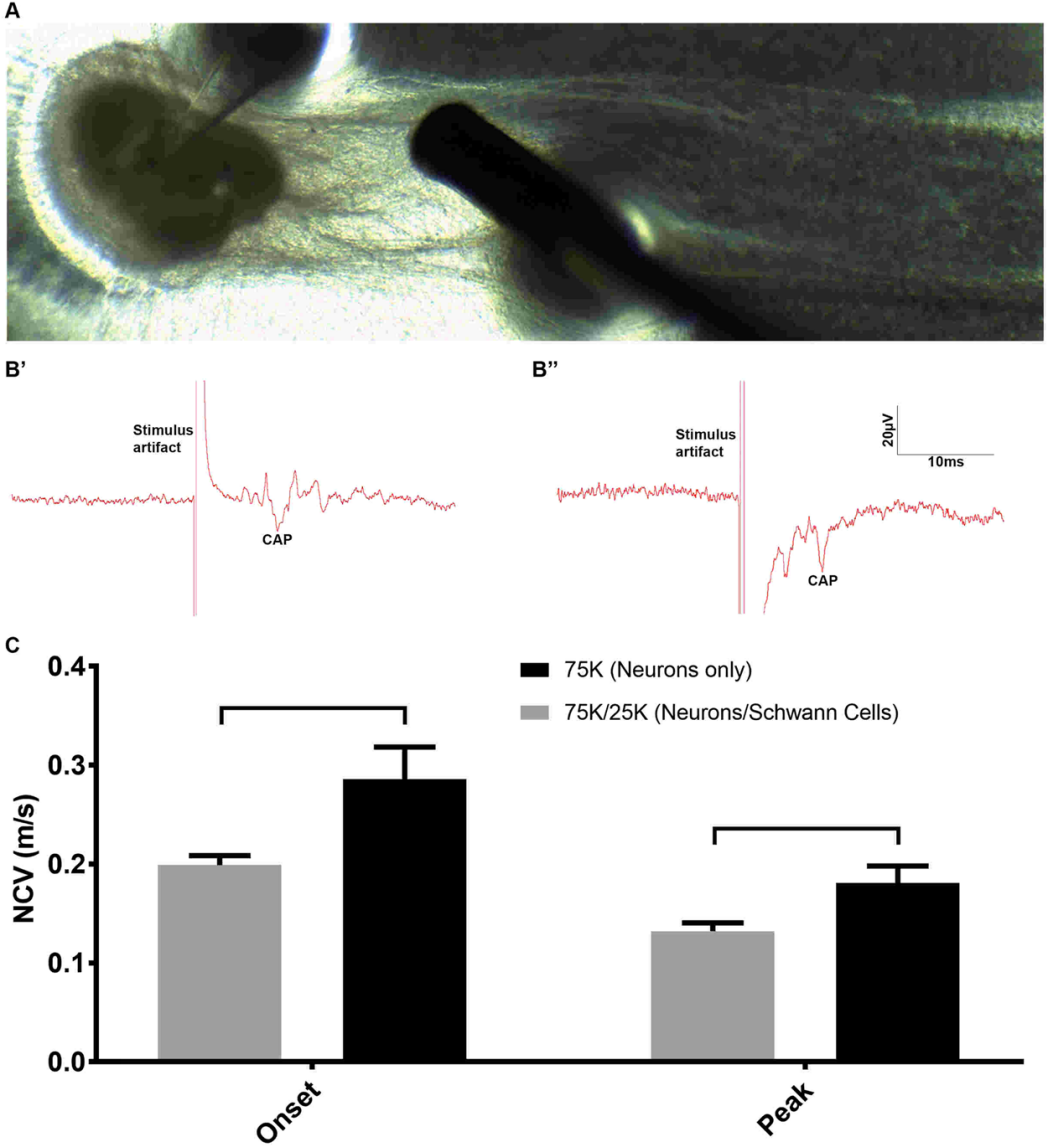
Electrophysiological testing of human nerve-on-a-chip (HNoaC). (**A**) Image showing the placement of stimulating and recording electrodes for recording compound action potentials (CAP) from *in vitro* HNoaC samples. (**B’**) Electrical trace showing the CAP generated in spheroid containing only 75,000 human neurons (hNs). (**B”**) Trace showing the CAP generated in a co-culture spheroid consisting of 75,000 hNs and 25,000 human Schwann cells (hSCs). (**C**) Graph showing a comparison of Onset and Peak NCV between hNs-only monoculture (75 K neurons) and co-culture (75 K hNs with 25 K hSCs). Onset NCV shows the start of the onset potential, while the Peak NCV shows the NCV for the biggest action potential. Nerve conduction velocity was observed to be slower in co-culture spheroids as compared to hNs-only spheroids (p-value ≤ 0.05). Peak and onset NCV were found to be not significantly different statistically. N = 3 & error bars represent the standard deviation (SD).

To determine whether we could measure NCV of hNs with or without hSCs, we used a technique similar to brain slice electrophysiology. We stimulated the axons inside the channel and recorded the compound action potential (CAP) from the cell bodies (Fig. 6A). Axons were stimulated about ~1–3 mm away from the cell bodies, and the distance the impulse traveled was calculated between the stimulating and recording electrodes. We evaluated two types of NCV, onset NCV and peak NCV, in order to determine the difference between the fastest signal and the peak signal (Fig. 6B’,B”). To our surprise, we found the onset and peak NCV with the hN/hSC co-culture samples was slower compared to hN mono-culture samples. Onset NCVs for 75 K hNs mono-cultures and 75 K/25 K hNs/hSCs co-cultures were determined to be 0.28 ± 0.07 and 0.20 ± 0.02 m/s, respectively, while the peak NCVs were found to be 0.18 ± 0.04 and 0.13 ± 0.02 m/s, respectively (Fig. 6C). We did not observe any significant differences in the peak and onset NCV. Onset and peak NCVs were difficult to measure from samples where the number of SCs were higher (co-cultures of hNs/SCs at 75 K/75 K and 75 K/50 K). Qualitative inspection of the samples revealed slightly less dense neurite outgrowth with co-culture samples, which can reduce the NCV.

## Discussion

In this study, we present the first 3D biomimetic, all-human *in vitro* model of peripheral nerve. To our knowledge this is the only organ-on-chip platform based entirely on human cells. Previous *in vitro* chip systems were primarily based on either rat DRGs^11^ or explanted rat nerve rootlets^13^. This microengineered dual hydrogel system retains the neuronal cell bodies in a defined location (i.e, the “ganglion”) and confines dense 3D axonal outgrowth within a narrow channel that extends linearly (i.e., the “fiber tract”) away from the clustered cell bodies. The system supports current “gold standard” functional (e.g., electrophysiological testing) and structural (e.g., qualitative and quantitative histological) endpoints. These are the preferred metrics for assessing neuropathological conditions associated with peripheral neuropathies, which represent a growing medical concern^3^. Through the evaluation of these clinically relevant metrics, this biomimetic human nerve could become an effective tool for screening new therapeutics and accessing their potential impact, whether neurotoxic or neuroprotective in nature. Innovative aspects of this study include the reproducible fabrication of neuron-Schwann cell co-culture spheroids, robust viability (~4 weeks), extensive neurite outgrowth (~5 mm) *in vitro*, and the effective myelination of hNs by hSCs. The ability to measure both quantitative and qualitative histology in combination with NCV in an *in vitro* setting is critical to predictive disease modeling, drug discovery, and toxicity screening.

### Challenges in producing *in vitro* nerve systems

*In vitro* myelination using primary hSCs has long been a challenge, due in part to complications associated with extracting hSCs from adult nerves^17,18^, contamination by fibroblasts^17–19^ and the transformation of SCs to a proliferative/non-myelinating phenotype *in vitro*^20,21^. Co-culture conditions are well established for myelination of rat DRG sensory neurons by embryonic, neonatal, and adult rodent SCs^22–24^. However, similar co-culture conditions fail to recapitulate myelination using human SCs cultured with rat DRG neurons^20^. Rigorous purification of primary human SCs or differentiation of human stem cells from human fibroblasts to SC-like cells results in limited levels of myelination of rat sensory neurons. However, the extent seen in the mixed-species cultures is significantly less compared to that achieved using embryonic rat SCs^20,25^, possibly due to species differences or density of SCs to axons. Recently, Clark, *et al*.^7^ successfully demonstrated myelination of human stem cell-derived sensory neurons by rat SCs. Still, an *in vitro* system exhibiting myelination of human iPSC-derived neurons by human Schwann Cells has remained elusive.

In the last several years, many studies focused on creating neuron-glia organoids to create brain-like tissue *in vitro*^26–30^. Interestingly, these strategies focused on differentiating aggregates of neural progenitor cells *in situ* into more defined neural structures. In contrast, we reverse-engineered the process by bringing together two differentiated cell types to evaluate their interactions with each other and the potential for self-assembly. To mimic the growth of dorsal root ganglia (DRG) *in vitro*, we produced neuron-Schwann cell spheroids using ultra-low attachment plates to facilitate crosstalk between axons and SCs, which is important for the differentiation of SCs toward a myelinating phenotype^31,32^; thus, by bringing axons and SCs close together in a 3D spheroid, we enhanced opportunities for cross communication and successful myelination. Following the addition of an anti-oxidant, ascorbic acid, we observed the first-ever evidence of myelination *in vitro* of induced pluripotent stem cell-derived human neurons by primary human Schwann cells. Both hNs and hSCs had different rates of self-assembly and spheroid fabrication individually. However, in combination the quality and speed of spheroid self-assembly improved considerably compared to the neuron-only condition. Based on spheroid diameter, co-culture spheroids were more compact compared to either hNs or hSCs monocultures, demonstrating enhanced interaction between the two cells types.

### Migration of schwann cells out of the spheroids

Schwann cell migration is a critical phenomenon during development and peripheral nerve regeneration following injuries^33^. Cues that direct the fate of neural crest cells to Schwann cell precursors and ultimately to Schwann cells are largely unknown; however, it has been understood for decades that both precursor cells and Schwann cells rely on growing axons for differentiation, proliferation and functional maturation^34^. Here, for the first time, we were able to observe this migration *in vitro* for tissues of human origin by creating a mini-ganglion comprised of hNs and hSCs. Based on the distance hSCs migrated relative to the length of axons in the channel, we think that hSCs primarily proliferate and migrate for the first week, until the NGF and L-ascorbic acid, factors known to enhance and mature myelination, were introduced to the system^35,36^. We believe that in the absence of FBS, hSCs do not proliferate or migrate along with growing axons and the limited interaction between hSCs in the bulb and axons in the channel results in the collapse of the inner gel. It was interesting to observe that hSCs only migrated to about ~1 mm outside the spheroid compared to total axonal growth of about 5 mm. This could be due to a lower ratio of hSCs to hNs during these experiments compared to typical 2D co-culture experiments, where the usual convention is to simply add a large number of Schwann cells in a smaller 2D area^7,37^. This relatively modest migration of hSC compared to axon outgrowth could also be a result of a limited pre-myelination culture period, followed by the addition of NGF to the media after the first week of growth. NGF has been shown to enhance neuron-Schwann cell interaction and also myelination^35^, and thus could be a factor which reduced the migration of SCs. Based on the migration of human SCs outside the spheroid, we hypothesize that this HNoaC model can also be used to study the migration potential of SCs in the presence of therapeutic molecules and thus create possible therapies for patients with peripheral nerve injuries or pathologies.

### Nerve structure

hSCs are known to behave differently *in vitro* to those sourced from rat in terms of their reactivity to mitogens and growth factors, as well as their failure to recapitulate myelination^38^. Our 3D spheroid model of human nerve exhibited features typical of nerve trunks observed during autopsy or biopsy procedures. Axons had a complete complement of organelles including cytoskeletal filaments and mitochondria, and often, but not always, were associated with sheaths of myelin characterized by closely approximated myelin laminae. The apposition of myelin layers varied among nerve fibers, and in some cases, laminar myelin formed in the absence of axons; both these findings are rarely encountered in differentiated nerves harvested *in vivo*, indicating that some differences in differentiation state do occur (as expected) in culture. That said, sufficient numbers of myelinated axons were observed in the mini-nerve portion of the co-culture system to render it a suitable surrogate for mixed somatic nerves (i.e., those containing densely myelinated, thinly myelinated, and unmyelinated axons).

### Evaluation of nerve conduction velocity

Different types of neuropathies are known for showing distinct neurophysiological characteristics^39^. In order to represent more than simply an incremental improvement over current models, *in vitro* microengineered nerve should be capable of defining electrophysiological changes conducive to conducting investigative and mechanistic toxicological studies. In this study, which is the first to use human iPSC-derived neurons to study population level electrophysiology, we were able to see differences in the NCV between the myelinated and unmyelinated human axons which shows that this system is sensitive enough to evaluate nerve function. To our surprise, myelinated hNs/hSCs co-culture samples showed a slower NCV compared to unmyelinated hNs-only mono-culture samples. A qualitative inspection of the culture revealed that the number of hSCs in the spheroid may have reduced the outgrowth and axonal density in the channel of HNoaC, which could result in a reduction of NCV. Also, because some axons appeared to turning back towards the spheroid in high SCs (50 K and 75 K, Supplemental Fig. 2) density co-cultures, we were not able to determine the optimal length between the point of stimulation and point of recording, which can impact the NCV calculations. However, we did not see this phenomenon in 75 K motor neuron alone & 75 K/25 K co-culture condition making the system useful for evaluating nerve physiology. Furthermore, the presence of non-neuronal cell bodies in co-culture spheroids may have decreased the probability of recording from the appropriately stimulated neuronal cell bodies. Importantly, NCV from hNs was found to be considerably lower as compared to the NCV values obtained in human patients^40–42^. This is not particularly surprising, considering we are utilizing an *in vitro* system, at room temperature, comprised of iPSC-derived neurons that are likely less mature as compared to myelinated axons of nerves assessed *in vivo*.

The elegant design of this fully HNoaC system unlocks new avenues in translational research. The platform can be used not only for understanding the mechanism of action for drug candidates on the basis of clinically relevant electrophysiological and histological metrics, but also for investigating basic mechanisms driving nerve pathologies, including but not limited to toxicity, demyelination, and other neurodegenerative conditions. In addition, comparison of data acquired in parallel using our Rat NoaC (RNoaC) platform^15^ and HNoaC for a given drug treatment or disease, could help further close the gap between nonclinical testing and our ability to anticipate responses and potential safety risks in humans.

## Conclusion

We have successfully created a biomimetic human cell based *in vitro* system which can provide gold standard physiological metrics associated with both clinical and *in vivo* neuropathology testing. First, we successfully fabricated spheroids comprising of hNs and hSCs, which then demonstrated long-term culture and robust neurite outgrowth in the dual-hydrogel system. Nerve conduction velocity was measured in the range of 0.13–0.28 m/s for these constructs. Morphological parameters were analyzed, demonstrating axonal diameter in the range of 0.4–0.55 µm, average g-ratio of 0.57, and percent axonal myelination of 4.7%. In the future, we will build on this current system by incorporating hSCs both in the bulb as well as the channel to enhance myelination. We plan to use this system for screening therapeutic molecules and studying various types of neuropathology, to further demonstrate the power of our HNoaC platform.

## Supplementary information

Supplementary Information

## Data Availability

The datasets generated during and/or analyzed during the current study are available from the corresponding author on reasonable request.
