# Supplementary material for: Engineering a 3D functional human peripheral nerve in vitro using the Nerve-on-a-Chip platform
Source: Sci Rep. 2019 Jun 20;9:8921. doi: 10.1038/s41598-019-45407-5 (PMC6586937; doi:10.1038/s41598-019-45407-5)
Supplement: Supplementary file 1 — Supplementary Information [file 41598_2019_45407_MOESM1_ESM.docx]

**Engineering a 3D functional human peripheral nerve *in vitro* using the Nerve-on-a-Chip platform**

Anup D. Sharma^1,*^, Laurie McCoy^1^, Elizabeth Jacobs^1^, Hannah Willey^1^, Jordan Q. Behn^1^, Hieu Nguyen^1^, Brad Bolon^4^, J. Lowry Curley^1^, Michael J. Moore^1,2,3,*^

AxoSim Inc.^1^, Dept. of Biomedical Engineering^2^, and Brain Institute^3^, Tulane University, New Orleans, LA.

GEMpath, Inc.,^4^ Longmont, CO, USA

*Corresponding authors

**Supplemental methods**

**Neurite outgrowth analysis -** Every 3^rd^ day during the growth period, images of the hydrogel constructs were captured using a Nikon phase contrast microscope with a 4X objective. The bulb portion of the construct was centered in the field of view. The z-plane of focus was adjusted such that neurites growing in the middle of the hydrogel were sharp, as opposed to the bottom Transwell® membrane or the top of the construct. The entire construct was imaged in three captures, and the images were stitched using the Fiji Pairwise Stitching function (Stephan Preibisch, Stephan Saalfeld, Pavel Tomancak; Globally optimal stitching of tiled 3D microscopic image acquisitions, *Bioinformatics*, Volume 25, Issue 11, 1 June 2009, Pages 1463–1465, <https://doi.org/10.1093/bioinformatics/btp184>.) A line was drawn across the widest portion of the bulb, perpendicular to the direction of growth. The 5 longest neurites were measured from this line. The average length and standard deviation were calculated and graphed in Prism GraphPad.

**Supplementary figures**


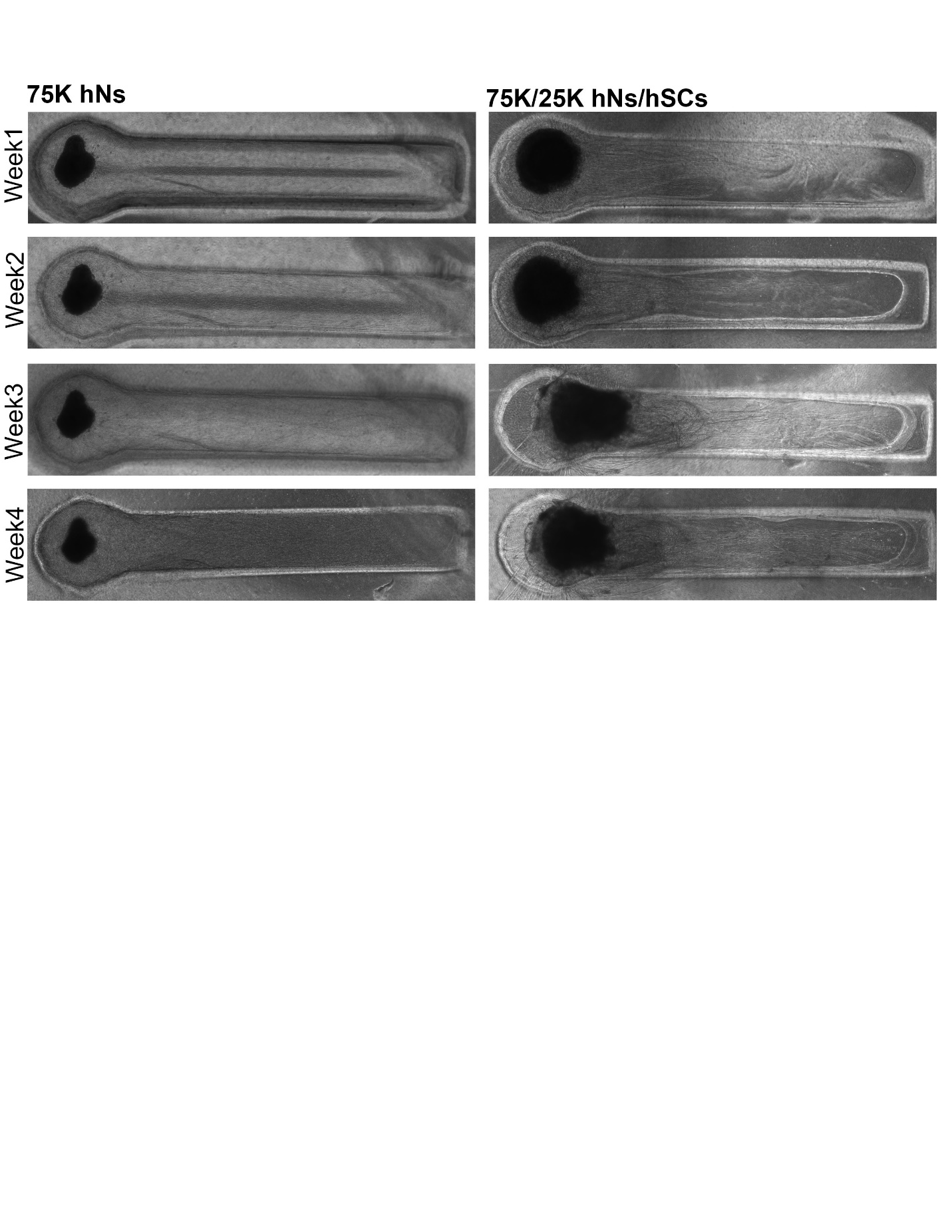


**Supplemental figure 1.** Images showing the growth of axons from a 75K human neuron (hNs)-only spheroid as compared to 75K/25K coculture spheroid consisting of hNs mixed with primary human Schwann cells. Axons grew ~1mm per week. With the coculture spheroid, axon tips started to turn back toward the spheroid after growing some distance.


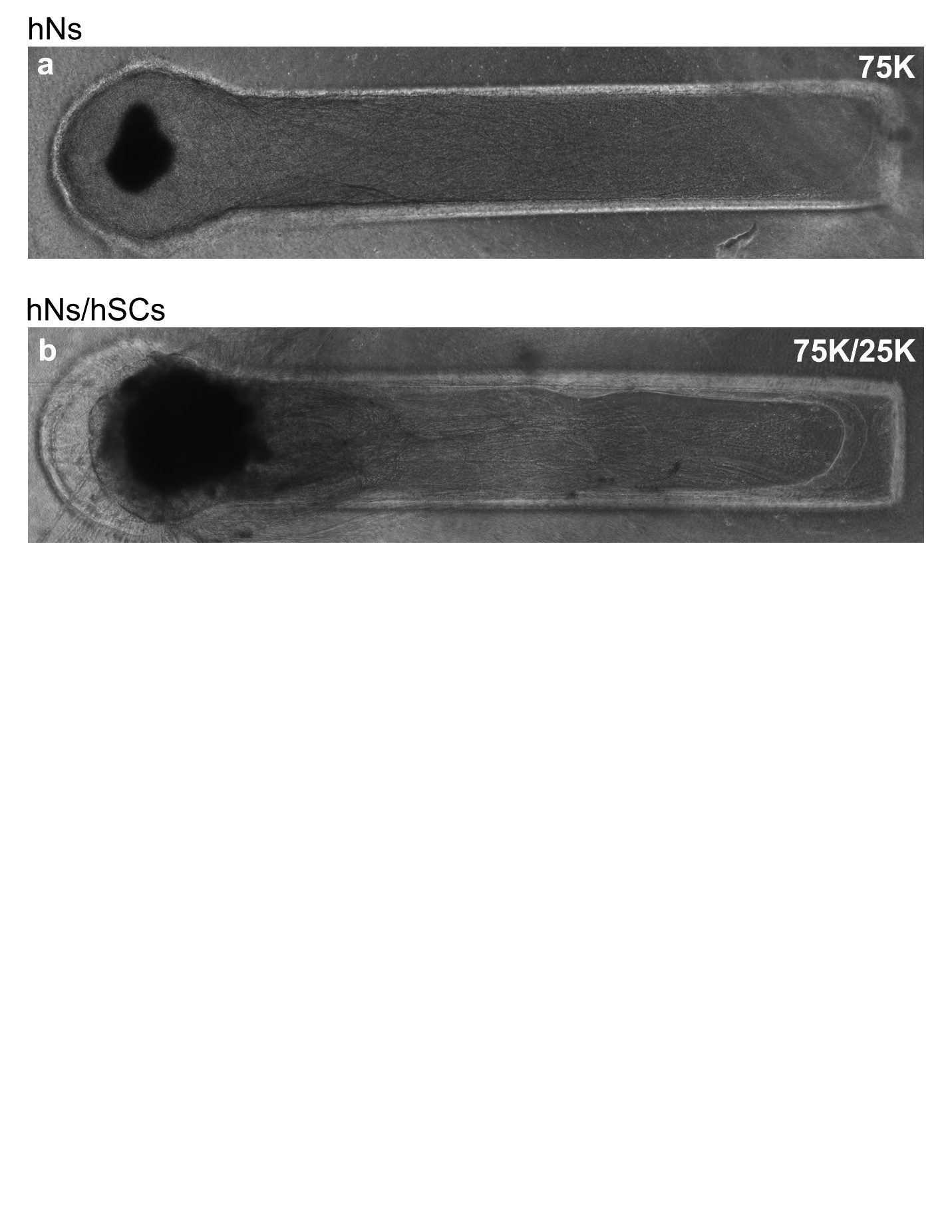


**Supplemental figure 2.** Representative images showing the growth of axons from a 75K human neuron (hNs)-only spheroid as compared to a 75K/25K coculture spheroid comprised of hNs and primary human Schwann cells just before electrophysiological testing (~4-week time point).


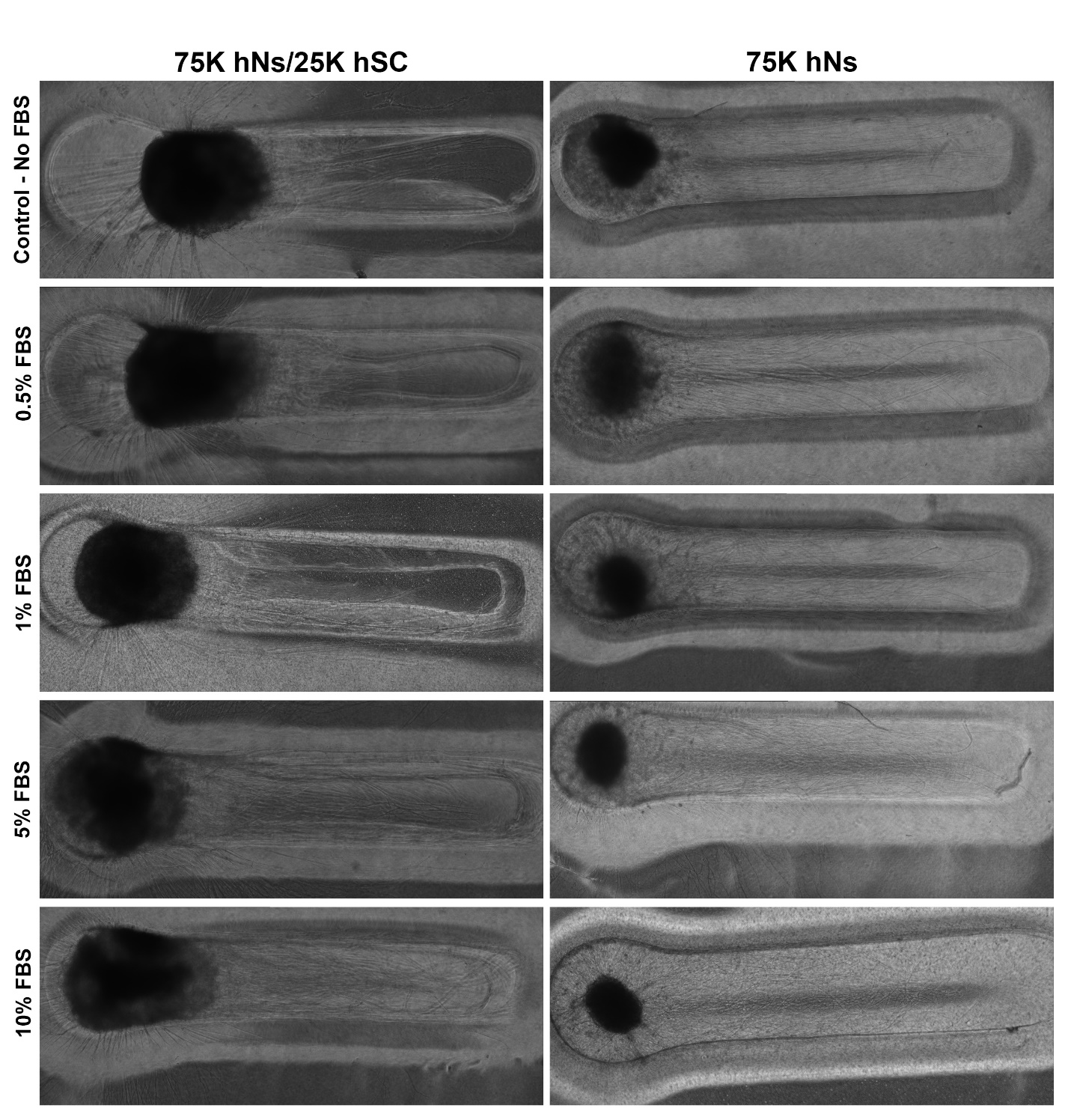


**Supplemental figure 3.** Impact of FBS on stabilization of Nerve-on-a-chip construct. An increasing amount of FBS impacted both the location of the spheroid in the channel as well as neurite outgrowth emanating from the spheroid. The important thing to observe was that FBS played a key role in the stabilization of coculture samples only while the motor neurons alone condition did not observe any influence from the increasing FBS concentration.


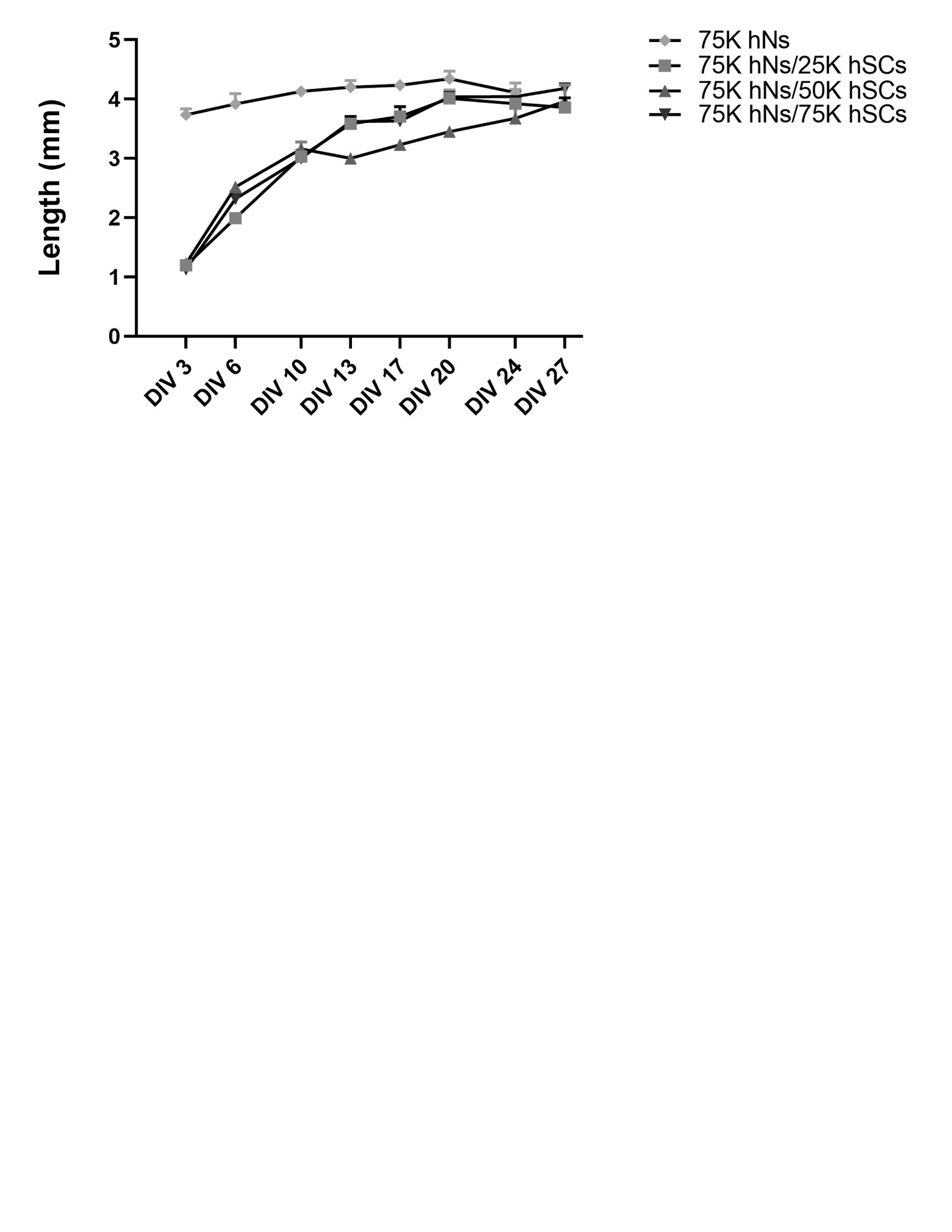


**Supplemental figure 4.** Axonal outgrowth observed in the NoaC system from different kind of mono and coculture spheroids. Monoculture spheroid saw a much faster neurite outgrowth compared to co-culture spheroids. Coculture spheroid had average neurite outgrowth of 3mm for the first two weeks which slowed down for the remaining two weeks.


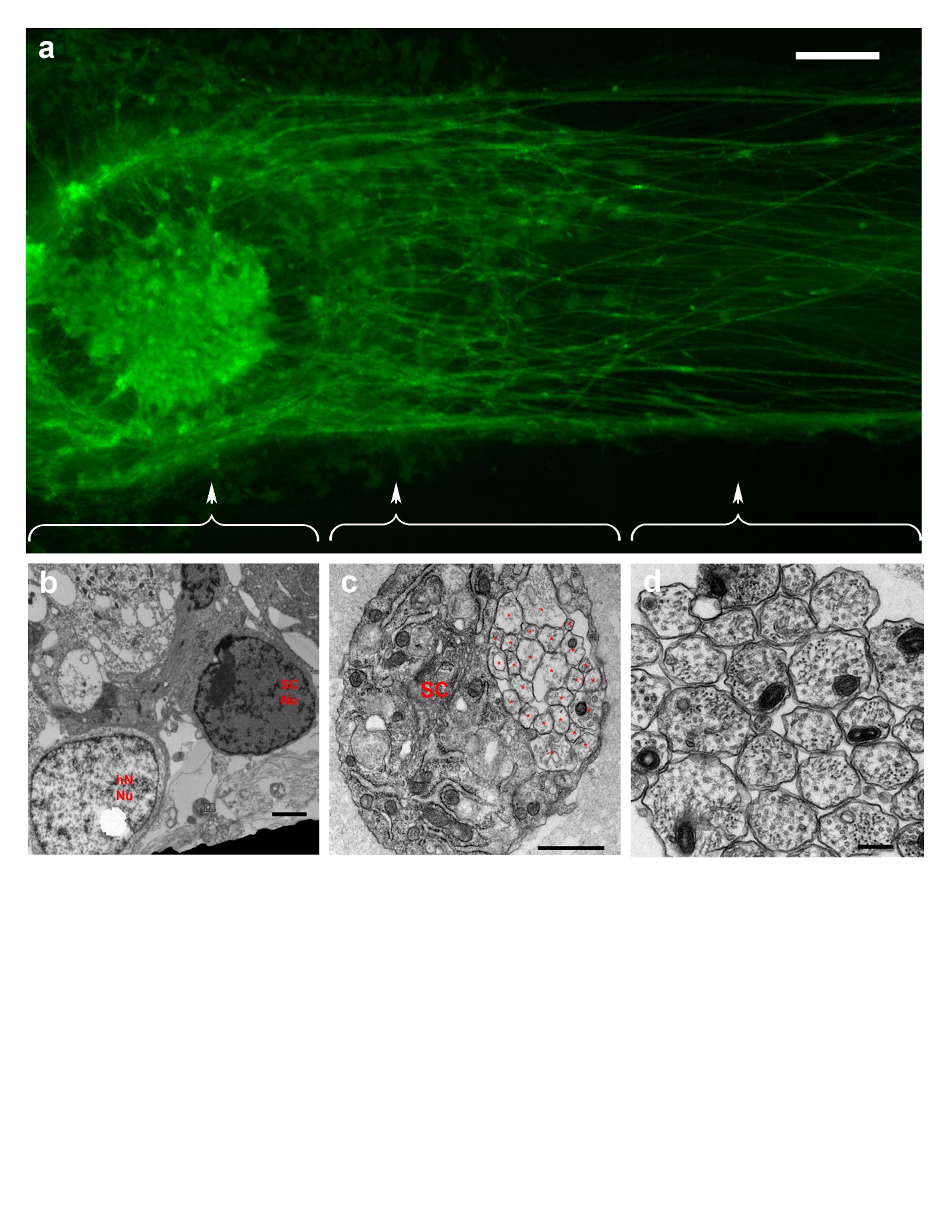


**Supplemental figure 5.** (a) Calcein staining of a coculture insert revealed that constructs were viable at the 4-week time point. Scale bar: 200µm. (b) TEM micrograph from bulb section revealed the presence of both neuron nuclei (hN Nu) and Schwann cell nuclei (SC Nu). Scale bar: 2µm. (c) Another section from the neck of the construct revealed a non-myelinating Schwann cell (SC) ensheathing many axons (*) creating the remak bundle. Scale bar: 1µm. (c) TEM micrograph from the distal region of channel revealed the presence of axons alone showing that neurons stayed inside the bulb region only. Scale bar: 0.2µm.
